# Development of a Classification System for Live Surgical Feedback

**DOI:** 10.1001/jamanetworkopen.2023.20702

**Published:** 2023-06-28

**Authors:** Elyssa Y. Wong, Timothy N. Chu, Runzhuo Ma, Istabraq S. Dalieh, Cherine H. Yang, Ashwin Ramaswamy, Luis G. Medina, Rafal Kocielnik, Seyedeh-Sanam Ladi-Seyedian, Andrew Shtulman, Steven Y. Cen, Mitchell G. Goldenberg, Andrew J. Hung

**Affiliations:** 1 Center for Robotic Simulation and Education, Catherine and Joseph Aresty Department of Urology, USC Institute of Urology, University of Southern California, Los Angeles; 2 Department of Urology, Weill Cornell Medicine, New York, New York; 3 Department of Computing and Mathematical Sciences, California Institute of Technology, Pasadena; 4 Thinking Lab, Department of Psychology, Occidental College, Los Angeles, California; 5 Department of Radiology, University of Southern California, Los Angeles

## Abstract

**Importance:**

Live feedback in the operating room is essential in surgical training. Despite the role this feedback plays in developing surgical skills, an accepted methodology to characterize the salient features of feedback has not been defined.

**Objective:**

To quantify the intraoperative feedback provided to trainees during live surgical cases and propose a standardized deconstruction for feedback.

**Design, Setting, and Participants:**

In this qualitative study using a mixed methods analysis, surgeons at a single academic tertiary care hospital were audio and video recorded in the operating room from April to October 2022. Urological residents, fellows, and faculty attending surgeons involved in robotic teaching cases during which trainees had active control of the robotic console for at least some portion of a surgery were eligible to voluntarily participate. Feedback was time stamped and transcribed verbatim. An iterative coding process was performed using recordings and transcript data until recurring themes emerged.

**Exposure:**

Feedback in audiovisual recorded surgery.

**Main Outcomes and Measures:**

The primary outcomes were the reliability and generalizability of a feedback classification system in characterizing surgical feedback. Secondary outcomes included assessing the utility of our system.

**Results:**

In 29 surgical procedures that were recorded and analyzed, 4 attending surgeons, 6 minimally invasive surgery fellows, and 5 residents (postgraduate years, 3-5) were involved. For the reliability of the system, 3 trained raters achieved moderate to substantial interrater reliability in coding cases using 5 types of triggers, 6 types of feedback, and 9 types of responses (prevalence-adjusted and bias-adjusted κ range: a 0.56 [95% CI, 0.45-0.68] minimum for triggers to a 0.99 [95% CI, 0.97-1.00] maximum for feedback and responses). For the generalizability of the system, 6 types of surgical procedures and 3711 instances of feedback were analyzed and coded with types of triggers, feedback, and responses. Significant differences in triggers, feedback, and responses reflected surgeon experience level and surgical task being performed. For example, as a response, attending surgeons took over for safety concerns more often for fellows than residents (prevalence rate ratio [RR], 3.97 [95% CI, 3.12-4.82]; *P* = .002), and suturing involved more errors that triggered feedback than dissection (RR, 1.65 [95% CI, 1.03-3.33]; *P* = .007). For the utility of the system, different combinations of trainer feedback had associations with rates of different trainee responses. For example, technical feedback with a visual component was associated with an increased rate of trainee behavioral change or verbal acknowledgment responses (RR, 1.11 [95% CI, 1.03-1.20]; *P* = .02).

**Conclusions and Relevance:**

These findings suggest that identifying different types of triggers, feedback, and responses may be a feasible and reliable method for classifying surgical feedback across several robotic procedures. Outcomes suggest that a system that can be generalized across surgical specialties and for trainees of different experience levels may help galvanize novel surgical education strategies.

## Key Points

**Question:**

Is there a reliable and generalizable method for classifying live surgical feedback that could be used to better understand what aspects of feedback may be associated with more optimal trainee responses?

**Findings:**

In this qualitative study of 29 surgical videos and 3711 teaching interactions, using the framework of trigger, feedback, and response was associated with reproducible categorization of feedback across surgeons of different skill levels and surgical cases.

**Meaning:**

This finding suggests that a feedback categorization system may reliably classify surgical feedback and be widely applied to various surgical specialties and procedures, which may help identify essential elements of the surgical teaching process.

## Teaser

This qualitative study assesses the reliability, generalizability, and utility of a method for classifying intraoperative feedback for trainees in surgery.

## Introduction

Interpersonal communication in the operating room (OR) is vital for safe surgery.^1,2^ During surgical training, verbal feedback between attending surgeon and trainee (whether fellow or resident), communicated with the intention of modifying trainee thinking or behavior,^3^ is important for improved performance and educational outcomes. Benefits associated with surgical feedback include improved intraoperative performance,^4,5,6^ accelerated technical skill acquisition,^6,7^ and increased resident autonomy.^8^ Despite these findings, there is no widely accepted system to categorize and evaluate such feedback, with associated improvements in feedback.

Some studies have sought to observe and categorize verbal feedback in the intraoperative setting. Previous publications have categorized teaching behaviors (eg, informing, questioning, responding, or tone setting)^9^ and described intraoperative communication (eg, explaining, commanding, and questioning).^10^ However, to our knowledge, a method of classifying and assessing surgical feedback that can be widely adopted to guide effective intraoperative learning has not been defined in the literature.

We sought to create a novel classification system capable of reliably characterizing surgical feedback while generalizing it to teaching interactions across surgical procedures. Further, we attempted to assess the utility of such a classification system to provide an understanding of what aspects of feedback may be associated with more optimal responses. Ultimately, we hope this line of research may allow surgical educators to optimize their feedback to trainees in the OR.

## Methods

### Data Collection

This qualitative study was approved by the University of Southern California. We recruited urological residents, fellows, and attending surgeons and obtained verbal consent from all participants. From April to October 2022, we prospectively recorded audio and video of robotic teaching surgeries in which a trainee actively controlled the robotic console for a portion of the surgery. This study followed the Standards for Reporting Qualitative Research (SRQR) reporting guideline.

Surgeons were categorized as trainers and trainees. Trainers were defined as those providing feedback, and trainees were those receiving feedback while actively operating on the console. Attending surgeons were always trainers, and residents were always trainees. Fellows were considered trainers when providing feedback to residents and trainees when receiving feedback from attending surgeons.

We used Open Broadcaster Software version 28.1.2 (Lain) to record synchronized video and audio. Video was streamed from the da Vinci Xi Robotic System (Intuitive) endoscopic camera view, and audio was captured with wireless microphones worn by surgeons.

Audiovisual recordings were manually time stamped for instances of feedback, defined as any dialogue intended to modify trainee thinking or behavior. Exact feedback quotations were transcribed. Dialogue with nonoperating residents or other OR personnel was excluded, as were teaching conversations that occurred while the attending surgeon was in active control of the robot.

### Development of the System

Our feedback classification system was developed using a grounded theory approach, which enabled us to remain open to all possible understandings and interpretations of the data. In addition, our methodology adopted a constructivist perspective, integrating medical education literature^9,10,11,12,13,14^ and experiences of our senior authors, who are engaged in medical education research and teaching in clinical and operative settings. These senior authors include an educational psychologist (A.S.) and 2 urologists (M.G.G. and A.J.H.).

**Figure 1.**
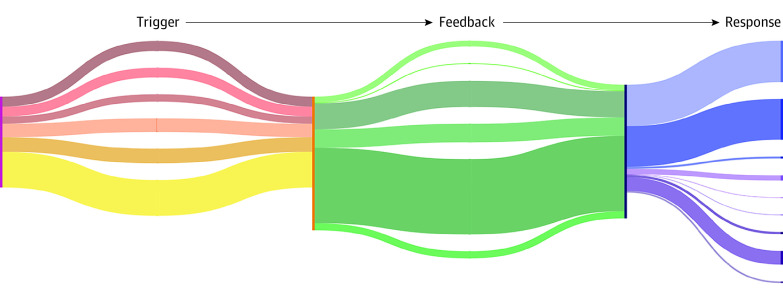
Coding 3711 Instances of Surgical Feedback An overview of the established types of triggers, feedback, and responses used to systematically code teaching interactions of 29 robotic procedures is presented. This figure does not represent all possible combinations of feedback types; for instance, anatomic and technical feedback may be provided in combination for the same line of feedback.

During initial data exploration, we identified recurring teaching themes and discussion points, from which we derived a schematic for feedback consisting of triggers, feedback, and responses (Figure 1). Detailed examples of this schematic being applied can be found in eAppendix 1 in Supplement 1. Our study team engaged in ongoing discussions to establish a catalog of repeating types of triggers, feedback, and responses (Table). For example, a warning type of trigger described an instance in which a trainee performed a bladder neck dissection but could not distinguish between prostate and bladder tissue. The following anatomic type of feedback was delivered in the form of “…look for [the] striated fibers of the detrusor muscle. That’s a warning sign you’re getting into the bladder.” Trainers may also have used a visual aid type of feedback and pointed directly with the telestration tool or tip of a suction-irrigation device if they were sitting at the bedside to draw a trainee’s attention to specific structures or details. The resulting trainee verbal acknowledgment type of response was demonstrated by the trainee reply of “Ah, okay.” A final data-recording schema was reached once it was possible to code an entire surgery with existing trigger, feedback, and response types. All surgeries were time stamped, transcribed, and coded with the same final classification system.

**Table.** Coding Types of Triggers, Feedback, and Responses

Feedback category	Type	Definition	Examples
Trigger	Error of omission	Behavior has occurred partially or not at all	“Keep cinching. Don’t stop”
			“You need to go more laterally”
	Error of commission	Incorrect behavior has occurred and has led to a definable mistake	“Never use coag[ulation] like that between 2 Wecks [clips]”
	Warning	Incorrect or suboptimal behavior has occurred, but there is no well-defined mistake	“Be careful there. You are getting close to an artery”
	Good trainee action	Trainee performs an acceptable action	A trainee finds the correct dissection plane and follows it carefully
	Trainee question	Trainee asks a question or makes a statement	“Do you prefer I take this en bloc?”
Feedback	Anatomic	Familiarity with anatomic structures and landmarks	“Stay in the correct plane, between the 2 fascial layers”
	Procedural	Pertains to timing and sequence of surgical steps	“You can switch to the left side now”
	Technical	Performance of a discrete task with appropriate knowledge of factors including exposure, instruments, and traction	“Buzz it”
	Visual aid	Addition of a visual element	Pointing with the suction cannula
			Using telestration on robotic console
	Praise	A positive remark	“Amazing job”
	Criticism	A negative remark	“It should never be like this”
Trainee response	Verbal acknowledgment	Verbal or audible confirmation by the trainee confirming that they have heard the feedback	“Okay, I see”; “Uh-uh, got it”
	Behavioral change	Behavioral adjustment made by the trainee that corresponds directly with the preceding feedback	Trainee immediately pulls more tightly on the suture thread after receiving feedback to cinch tightly
	Ask for clarification	Trainee asks for feedback to be restated due to lack of understanding	“What do you mean?”
Trainer response	Approval	Trainer verbally demonstrates that they are satisfied with the trainee behavioral change	“Yes”; “Mhm”
	Disapproval	Trainer verbally demonstrates that they are not yet satisfied with the trainee behavioral change	“No”; “Try again”; “Not quite”
	Repeats feedback (identical)	Trainer repeats the same feedback regarding the same step of procedure and anatomy	Trainer: “Stay in the correct plane”
			Trainer (1 min later): “Stay in the junction”
	Repeats feedback (similar)	Trainer gives similar feedback to a previous instance but varying based on its anatomic, procedural, or technical component	Trainer: “Stay in the correct plane”
			Trainer (30 min later): “Stay in the correct plane”
	Takes over (for safety)	Trainer takes control of the robot out of concern for patient safety	Trainer takes control of the console because the trainee is unable to identify the bleeding vessel and get proper hemostasis
	Takes over (for nonsafety reason)	Trainer takes control of the robot	1) To perform quality-control assessment To perform a step of the procedure that is beyond the trainee’s scope of ability To help reorient the trainee To demonstrate

### Reliability of the System

Time stamping, transcribing, and coding were performed by 3 trained research assistants (E.Y.W, I.S.D, and C.H.Y.). To assess interrater agreement, each rater viewed the same full-length robotic simple prostatectomy surgery (ie, the training surgery). This surgery was selected given that it contained a broad sampling of different types of triggers, feedback, and responses. It included 2.5 hours of surgical footage and 195 total instances of feedback. The 3 raters independently time stamped, transcribed, and coded established types of triggers, feedback, and responses in this surgery. Interrater agreement for each step was calculated using prevalence-adjusted and bias-adjusted κ (PABAK), with a κ of more than 0.6 considered to indicate moderate to substantial agreement.^15^

Rater discrepancies were discussed until a consensus was reached. To ensure consistent coding, raters co-authored a codebook defining each type of trigger, feedback, and response, with examples. This shared codebook served as a reference for all raters (eAppendix 2 in Supplement 1).

### Generalizability of the System

We compared the feedback received by fellows vs residents given that this training distinction can exist across institutions and surgical specialties. First, instances of feedback were grouped by whether the trainee was a fellow or a resident. To accommodate the nested data structure of multiple individuals contributing varying numbers of triggers, feedback, and response types within each group, we used a hierarchical Poisson model with generalized estimating equations (GEE) and a natural log link function. We estimated weighted mean rates (ie, prevalence rate) of each type of trigger, feedback, and response from the hierarchical GEE model for the combined fellow group and resident group. Prevalence rate ratios (RRs) of each type of trigger, feedback, and response for residents vs fellows were determined using the log link function for modeling, which was interpreted as relative risk. We investigated the overdispersion assumption through the goodness-of-fit test using the Pearson χ^2^ to degrees-of-freedom ratio using a negative binomial distribution when the ratio was larger than 1 (with a threshold of 1.5).

We next classified all instances of feedback according to 2 types of general surgical activity: suturing (eg, vesicourethral anastomosis during prostatectomy) vs tissue dissection (eg, seminal vesical dissection during prostatectomy). Suturing and dissection were chosen for their ubiquity across surgical procedures. Videos were marked for the start/stop time of each procedural step based on our institution’s robotic training curriculum.^16^ Instances of feedback during suturing steps were aggregated and compared with feedback occurring during dissection steps. For these 2 groups, a hierarchical GEE model was used to determine the prevalence rate of each type of trigger, feedback, and response. We again used RR to compare the frequency of each type of trigger, feedback, and response for dissection vs suturing tasks.

### Utility of the System

Our classification system accounted for recurring types of responses to feedback. Because we were interested in studying feedback that was useable, easily understood, and associated with changes in outcomes, we further examined instances of feedback that were associated with obvious trainee behavioral change and verbal acknowledgment. Trainee behavioral changes were exemplified by trainees immediately modifying their behavior to address the feedback given to them. For example, feedback to “retract more” was followed by a trainee applying increased traction. Trainees also used verbal acknowledgment to signal that they understood the feedback. This included succinct expressions like “okay” and “yeah.”

In some instances, trainers explicitly voiced satisfaction with a trainee response. A trainee who properly corrected his or her dissection technique might be immediately told, “That’s a much better sweeping motion,” which served as a trainer approval response.

### Statistical Analysis

Associations between feedback types and responses were also examined using a hierarchical GEE model. The 2-way interaction term was added to the model to explore the interaction between any pair of feedback types in association with response. Statistical significance was set at *P* < .05, and all hypothesis tests were 2-tailed. SAS statistical software version 9.4 (SAS Institute) was used for all data analysis.

## Results

In total, 29 robotic teaching surgical procedures were recorded, with 3711 instances of feedback across 69 hours and 17 minutes of surgical recordings (a mean of 128 instances per case). Participants included 4 attending surgeons, 6 fellows, and 5 residents (postgraduate years, 3-5).

### Reliability of the System

After standardized training, 3 raters had substantial agreement on the presence and timing of feedback within the training case (191 of 195 instances [98.0%] and 184 of 195 instances [94.4%] for rater pairs E.Y.W.-C.H.Y and E.Y.W.-I.S.D, respectively). Rater pairs had a moderate to substantial agreement on identifying the same 5 types of triggers (PABAK minimum, 0.56 [95% CI, 0.45-0.68] to maximum, 0.91 [95% CI, 0.85-0.97]), 6 types of feedback (PABAK minimum, 0.70 [95% CI, 0.60-0.80] to maximum, 0.98 [95% CI, 0.95-1.00]), and 9 types of responses (PABAK minimum, 0.62 [95% CI, 0.48-0.71] to maximum, 0.99 [95% CI, 0.97-1.00]).

### Generalizability of the System

We evaluated our system on 29 robotic surgical procedures at our institution. This included 6 different types of procedures: radical prostatectomy (12 surgical procedures), simple prostatectomy (8 surgical procedures), radical nephrectomy (5 surgical procedures), partial nephrectomy (1 surgical procedure), nephroureterectomy (2 surgical procedures), and inguinal hernia repair (1 surgery).

**Figure 2.**
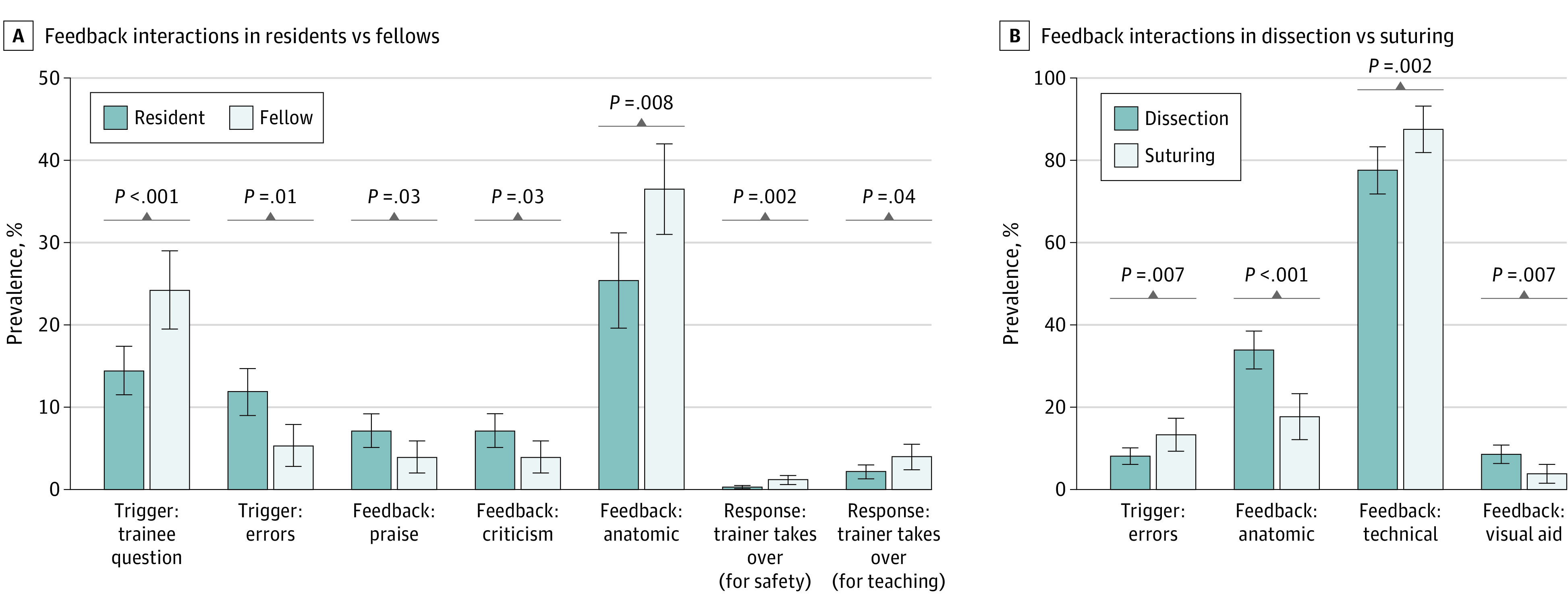
Feedback by Trainee Experience Level and Surgical Task Error bars indicate 95% CIs.

Feedback was assessed by trainee surgical experience level (Figure 2A). Residents made errors that triggered feedback more often than fellows (RR, 2.22 [95% CI, 1.62-2.83]; *P* = .01). Residents received more praise (RR, 1.82 [95% CI, 1.33-2.33]; *P* = .03) and criticism (RR, 1.82 [95% CI, 1.33-2.33]; *P* = .03) types of feedback than fellows.

Fellows elicited feedback more often by asking questions compared with residents (RR, 1.68 [95% CI, 1.41-1.94]; *P* < .001). Fellows received more anatomic feedback than residents (RR, 1.44 [95% CI, 1.17-1.70]; *P* = .008). As a response, attending surgeons took over for safety concerns more often for fellows than residents (RR, 3.97 [95% CI, 3.12-4.82]; *P* = .002). Attending surgeons also took over for non–safety-related teaching (eg, show-and-tell teaching purposes) more frequently for fellows compared with residents (RR, 1.81 [95% CI, 1.19-2.38]; *P* = .04).

Feedback was also assessed across varying surgical tasks (suturing vs tissue dissection) (Figure 2B). Suturing involved more errors that triggered feedback than dissection tasks (RR, 1.65 [95% CI, 1.03- 3.33]; *P* = .007). Trainees received more anatomic feedback from trainers when performing dissection tasks (RR, 1.92 [95% CI, 1.61-2.22]; *P* < .001) and more technical feedback when suturing (RR, 1.13 [95% CI, 1.04-1.25]; *P* = .002). Visual aid (ie, telestration) was used more often in conjunction with verbal feedback during dissection tasks vs suturing (RR, 2.27 [95% CI, 1.73-2.94]; *P* = .007).

### Utility of the System

Trainer feedback was received with explicit trainee behavioral change or verbal acknowledgment in most instances (2819 feedback instances [75.9%]). Fellows exhibited these responses significantly more than residents (RR, 1.11 [95% CI, 1.04-1.18]; *P* = .002). As a secondary response, a trainer explicitly approved the trainee change in 558 of these 2819 instances (19.8%) or explicitly disapproved the trainee’s change in 66 instances (2.3%). Fellows and residents received trainer explicit approval and disapproval to similar degrees (fellows: RR, 1.00 [95% CI, 0.73-1.28]; *P* = .98; residents: RR, 1.23 [95% CI, 0.49-1.97]; *P* = .59).

**Figure 3.**
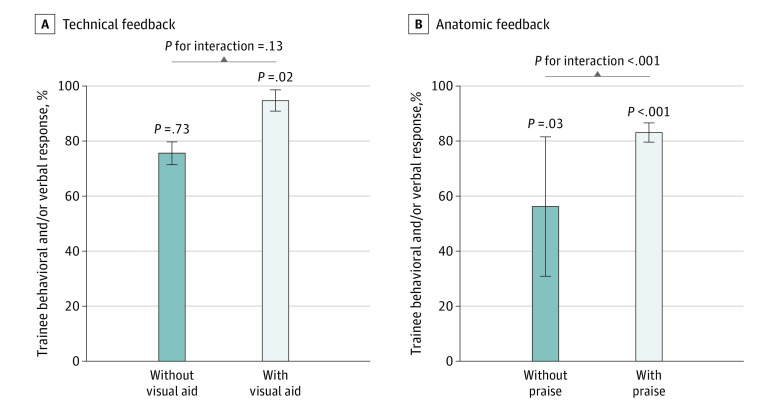
Interaction Between Types of Feedback and Responses Error bars indicate 95% CIs.

Technical feedback provided with a visual aid component (eg, the trainer saying, “Take a bigger bite so you get some mucosa” while simultaneously pointing with the telestrator at the mucosal edge) was found to have a significantly increased rate of trainee behavioral change or verbal acknowledgment responses compared with no technical feedback (RR, 1.11 [95% CI, 1.03-1.20]; *P* = .02). Providing technical feedback without visual aid did not have a statistically significant association with trainee behavioral change or verbal acknowledgment responses (RR, 1.01 [95% CI, 0.93-1.10]; *P* = .73). The difference between the RR of technical feedback with visual aid and the RR of technical feedback without visual aid was not statistically significant (Figure 3A).

Anatomic feedback with a component of praise (eg, “Amazing work on the posterior [plane]”) was associated with increased rates of trainee behavioral change or verbal acknowledgment responses compared with no anatomic feedback (RR, 2.76 [95% CI, 2.28-3.24]; *P* < .001). Giving anatomic feedback without praise was also found to have a significantly increased rate of trainee behavioral change or verbal responses compared with no anatomic feedback (RR, 1.05 [95% CI, 1.01-1.10]; *P* = .03). The difference between the RRs of anatomic feedback with and without praise was statistically significant (Figure 3B).

## Discussion

This qualitative study introduces an objective classification system for live surgical feedback that was associated with reliable outcomes and may be generalized to live teaching interactions across different surgical procedures, trainee experience levels, and surgical tasks. We found the potential utility of such a system by evaluating the association of certain feedback types with greater changes in trainee behavior. Establishing a systematic approach to studying intraoperative surgical feedback is essential to improving learning interactions in the OR.

### Reliability of the System

Evaluating reliability and consistency is challenging given the heterogeneity of feedback. Hauge et al^9^ previously reported the reliability of a feedback instrument with Cronbach α values ranging from 0.21 to 1.00 (median, 0.85) between 2 raters evaluating 1169 teaching behaviors. Considering such published standards, we achieved moderate to substantial agreement in identifying various types of triggers, feedback, and responses established by our classification system. In our user experience, frequent discussion among raters and the development of a shared codebook was essential to establishing higher reliability of the feedback classification tool.

### Generalizability of the System

By observing 29 surgical procedures and 6 different types of procedures, we created a feedback classification system informed by the largest annotated sample of surgical feedback to our knowledge, with the goal of making it generalizable across surgical disciplines. Using our classification system, we detected explainable differences in feedback associated with trainee experience level and surgical task. Our analysis found that fellows received greater amounts of anatomic feedback than residents. This may be associated with the increased time that fellows spent on complicated dissection steps involving granular identification of key structures. By our count, attending surgeons also took over due to safety concerns more often with fellows. It is plausible that this finding was associated with the increased level of challenge purposefully given to fellows as they worked closer to the limits of their capabilities.

We initially explored live feedback in robotic surgery owing to the convenience with which surgical footage could be recorded. Future studies may incorporate different surgical approaches, such as open, laparoscopic, endoscopic, and other surgical specialties to assess whether quantitative and explainable differences in triggers, feedback, and responses may be identified in other settings.

### Utility of the System

While other studies have proposed different methods of cataloging feedback,^9,10^ we did not find any studies using comparative analysis. In applying our classification system and subsequent analysis, we found that feedback combinations were associated with responses. We found that pairing anatomic feedback with praise was associated with increased rates of trainee behavioral change or verbal acknowledgment responses compared with anatomic feedback without praise. These early-stage findings may provide proof of concept for our classification system. Our methodology may make feedback data compatible with quantitative analysis, which has never been demonstrated, to our knowledge. With the addition of more surgical procedures to our sample, we may investigate feedback combinations associated with rarely occurring responses, such as trainer takes over for safety. Our observations may also serve as a basis for future dry laboratory studies in which we modify selected combinations and validate their associations with trainee performance.

Additionally, our feedback classification system may potentially bridge communication gaps between trainers and trainees.^17,18^ Fundamental differences in how trainers and trainees perceive, comprehend, and address information may make it difficult for trainers to understand difficulties trainees face. While a trainer may view a situation as straightforward, a trainee may find it cognitively taxing. Our feedback classification system has the potential to inform trainers how trainees respond to their feedback. Meanwhile, trainees may glean insight into the feedback they most commonly receive. With increased follow-up time, teaching interactions may also be examined over time as trainer-trainee pairs continue to work together or new pairings form.

Surgical feedback is inherently difficult to study owing to its unstructured and individualized nature. However, quantifying and analyzing feedback systematically is crucial to the overarching goal of optimizing feedback and improving surgical training. With a new methodology for classifying surgical feedback, there is a possibility of analyzing patterns and discovering which feedback combinations may be associated with increased or decreased rates of certain responses.

### Limitations

There are several limitations to our study. This study was performed at a single institution; however, it still represents the largest annotated repository of surgical feedback, to our knowledge. While data were collected within a single specialty (urology), the diversity of teaching surgical procedures observed required an array of technical and nontechnical skills that are fundamental to surgery in general. Additionally, our study relied on some degree of subjective interpretation of the feedback by third-party, nonsurgeon raters. To temper this, all raters underwent a regimented training process and reached moderate to substantial interrater reliability before coding surgical cases for analyses.

We are mindful that our classification system is not yet equipped to make value judgments regarding the quality of feedback. Trainee and trainer responses that we observed were designed to capture immediate actions that followed feedback. We caution against using them to make direct inferences about the quality of feedback (ie, associated with a good or bad response). Additionally, trainer approval and disapproval responses we captured were limited to instances in which trainers explicitly voiced satisfaction or dissatisfaction. Further work may explore instances of feedback in which trainers responded with silence.

## Conclusions

This qualitative study’s novel systematic classification for live intraoperative feedback delineates current educational interactions in the OR. Implementing a system that can reliably characterize surgical feedback and be widely generalizable to various surgical specialties and procedures may further uncover essential elements of the surgical teaching process, which may ultimately be associated with optimized intraoperative education.

## Notes

Supplement 1. **eAppendix 1.** Applying Feedback Classification System to Clinical Examples **eAppendix 2.** Codebook Shared Among Raters

Supplement 2.
Data Sharing Statement
