# Supplementary material for: Development of a Classification System for Live Surgical Feedback
Source: JAMA Netw Open. 2023 Jun 28;6(6):e2320702. doi: 10.1001/jamanetworkopen.2023.20702 (PMC10308254; doi:10.1001/jamanetworkopen.2023.20702)
Supplement: Supplement 1. — eAppendix 1. Applying Feedback Classification System to Clinical Examples eAppendix 2. Codebook Shared Among Raters [file jamanetwopen-e2320702-s001.pdf]

Supplemental Online Content

Wong EY, Chu TN, Ma R, et al. Development of a classification system for live surgical feedback. *JAMA Netw Open*. 2023;6(6):e2320702.
doi:10.1001/jamanetworkopen.2023.20702

eAppendix 1. Applying Feedback Classification System to Clinical Examples
eAppendix 2. Codebook Shared Among Raters

This supplemental material has been provided by the authors to give readers additional information about their work.

© 2023 Wong EY et al. *JAMA Network Open.*

eAppendix 1. Applying Feedback Classification System to Clinical Examples

Example 1:
A Trigger is defined as any action by the operating trainee (either verbal or nonverbal) which prompts the trainer to provide Feedback. As illustrated in the following example, the trainee's inefficient use of an instrument triggers the trainer to provide guidance:

> ***Situation:*** *The trainee is in control of the robot and performing lymph node dissection. After opening the peritoneum, the trainee works on creating a space to visualize the deeper lymph node packets. They use cold scissors to dissect sharply.*

The trainee's ineffective technique is coded as an *error of omission* Trigger. This is due to the fact the trainee is attempting to perform the correct behavior (dissection) but is missing the proper technique.

> ***Trainer***: "If you snip a little at a time it will take you forever. Just blunt dissect and open it up. Push medially and be a little more aggressive there. You can see the bone, right? You can push medially; there's nothing there, okay?"

This ensuing verbal dialogue given by a trainer is the Feedback, with the intention of modifying the trainee's understanding and behavior. The trainer provides a combination of *technical* Feedback (blue) and *anatomic* Feedback (orange).

> ***Trainee****: "*Ah, okay, yes."

> *The trainee closes the scissors and begins to push medially.*

> ***Trainer:*** **"**Exactly like you're doing. Okay."

In Response to the Feedback, the trainee verbalized understanding and adjusted his technique. The trainer also affirms that appropriate corrective behavior has been achieved. Feedback can lead to one or more Responses. Responses are the verbal and/or behavioral reactions directly resulting from the trainer's Feedback. In this example, the Feedback led to two Responses from the trainee, one verbal and one behavioral. Having witnessed the correction in technique, the trainer gave an *approval* Response that could be traced directly back to the original Trigger and Feedback.

Example 2:

> ***Situation:*** *The trainee is in control of the robot and performing a right radical nephrectomy. The trainee is reflecting the mesenteric fat away to gain access to the retroperitoneum. The trainer is scrubbed at the bedside with the suction-irrigation device. The trainee uses the gasper to spread apart tissue planes.*

In this scenario, the trainee has already started dissecting in the incorrect plane, which is coded as an *error of commission* type of Trigger.

> ***Trainer****:* "Wait hang on for a second. You just went a layer too deep. You see? This is the [perinephric] fat."
>
> *The trainer taps perinephric fat with the tip of the suction device*
>
> "And this is the layer you want"
>
> *The trainer uses the tip of the suction device to lift the mesenteric fat*
>
> "It's going to be very sticky because of the infections she's had"

The trainer uses a combination of *anatomic* (orange), *visual aid* (purple), and *procedural* (red) Feedback. Here, the suction device is used as a pointer, providing a *visual aid* element. The trainer also provides procedural Feedback which considers the patient's medical history and anticipates the possible difficulties that lay ahead.

> *The trainee moves their grasper next to the trainer's suction device and follows the natural tissue plane.*
>
> ***Trainer:*** **"**Yes, yes."

In Response to Feedback, the trainee demonstrates a *behavioral change* by adjusting the depth of their dissection. Furthermore, the trainer gave an *approval* Response.

eAppendix 2. Codebook Shared Among Raters

Our feedback classification system uses the schema of Trigger, Feedback, and Response. However, this document is written in the order of operations that our trained raters found to be helpful when in the process of timestamping, transcribing, and coding. Our raters all contributed to the development of this codebook and frequently revisited it to ensure internal consistency throughout the study.

I. Timestamping and Transcribing Feedback

A. To be considered Feedback, dialogue must meet the following criteria
1. A trainer is talking directly to the trainee actively operating (i.e. controlling the robot)
2. Communicated with the intention of modifying the trainee's thinking or behavior

B. Exclude the following:
1. Personal conversations
2. Trainer talking to surgical techs/trainee(s) who are not actively operating (this could be another trainee at the bedside)

C. A timestamp should be recorded at the start of meaningful Trainer dialogue
1. For example, a trainer gives the following Feedback: "So… [5-second pause] instead, I would do…"
   Timestamp the beginning of "Instead I would do…"
2. Use 00:00:00 format

D. A new instance of Feedback should be created if
1. A Response occurs
2. A new Trigger occurs
3. For example, a trainee is given Feedback to "buzz that." Before they can respond, they make another error which Triggers new Feedback. Because a new Trigger has occurred, a new instance of Feedback should be timestamped and transcribed. Consider whether the Trainee eventually addresses the first Feedback to "buzz that."

E. Transcribe the exact Feedback given by the Trainer

II. Triggers

A. These are event(s) that prompt feedback. They can be verbal or nonverbal

B. There can be more than one Trigger for an instance of Feedback

C. It can be helpful to use the Feedback to determine what the Trigger is

D. *Error of omission*: when a behavior has *not* occurred (i.e. inaction) or *not enough* of behavior has occurred.
1. Example Feedback: "Coag that" (in this scenario the trainee failed to coag in a timely fashion)
2. Example Feedback: "Go more lateral," "keep cinching" (in these scenarios the trainee performed some action, but not enough of it)

E. *Error of commission*: an incorrect behavior occurred and led to a definable mistake
1. Example: the trainee uses improper technique and is given Feedback to "never use coag like that between two wecks."

F. *Warning*: an incorrect or sub-optimal behavior has occurred but there is an absence of a well-defined mistake
1. The most obvious example is when the trainee is told "be careful"
2. Example: the trainee is given Feedback "don't cut into the yellow fat" but there is no evidence they have done so yet.

G. *Good trainee action*: the trainee performs an acceptable action
1. When the Trigger is a good trainee action, often the Feedback will reflect this: "Good, that is the correct plane you should be in."

H. *Trainee question*: the trainee asks a question or makes a statement that prompts Feedback

© 2023 Wong EY et al. *JAMA Network Open.*

1. Example: “Do you want me to take this en bloc?”
2. Example: “I’m not sure where the ureter is yet”

III. Feedback

A. *Anatomical*: discussing anatomic structures and location/directionality. Of note, the exact anatomical name may not be said by the trainer.
   1. Examples:
      “Stay in the correct plane, between the two fascial layers”
      “Stay in the [prostate] capsule”
      “You're in the right plane” (i.e., the right tissue dissection plane)
      “You're in the right area”

B. *Procedural*: pertains to timing and sequence of surgical steps, decision making, situational awareness, and awareness of potential/general areas of complications
   1. Examples:
      “Finish what you're doing here first, then move on”
      “You can switch to the left side [now]”
      “What was the patient’s hemoglobin?”

C. *Technical*: performance of a specific/discrete task. Appropriate use and knowledge of exposure, instruments, traction, and energy (e.g. positioning and handling of instruments and tissue).
   1. Examples: “Buzz it,” “cold cut,” “gentle,” “careful,” “fix your retraction,” or “use your other arm”

D. *Visual aid*: feedback using a visual element. This can be the telestrator (which looks like a blue pointer) or the tip of the suction-irrigation device if the trainer is assisting at the patient's bedside

E. *Praise*: clearly positive remarks
   1. Examples: “Amazing job,” “good work”
   2. Exclude phrases that are less clearly positive such as “okay fine”

F. *Criticism*: clearly negative remarks (these are remarks which go beyond factual statements)
   1. Example: “You just ripped the tissue off, that’s terrible” would be considered *criticism* versus “Be careful, you are damaging the tissue” is factual and less of a personal attack

IV. Responses

A. *Trainee verbal acknowledgment*: trainee gives verbal/audible confirmation that they have heard the feedback. This includes minor verbalizations like “Mhm.”

B. *Trainee behavioral change*: trainee makes a behavioral adjustment that reflects the preceding feedback. This does not mean that the trainee must make a perfect correction. Any attempt to address the Feedback with a behavioral change counts.
   1. Example: a trainer gives the Feedback “retract more with your fourth arm.” The trainee subsequently grabs the tissue and applies increased traction.

C. *Trainee asks for clarification*: trainee asks the trainer to restate the Feedback because they do not understand or asks for further details to be provided.
   1. Do not code if the trainee asks for repetition because they are simply unable to hear Feedback.
   2. Example: trainee says, “I’m not sure I understand…” or “what do you mean you want me to use the vessel loop?”

D. *Trainer approval*: trainer verbally demonstrates that they are satisfied with the *trainee’s behavioral change* Examples: “that’s good,” “that’s fine,” “ok”, “yup”, “uh-huh”
      If a trainer says “yup” independent of a previous line of Feedback/with a distinctly different trigger, do not code this as *trainer approval*.

E. *Trainer disapproval*: trainer verbally demonstrates they are not yet satisfied with the trainee’s behavioral change
   1. Examples: a trainer says “no", "try-again", or "not quite"

© 2023 Wong EY et al. *JAMA Network Open.*

F. *Trainer Repeats Feedback (identical)*: trainer repeats Feedback with the same meaning during the same step of the procedure and in the same anatomical region
   1. The trainer can use the same Feedback verbatim or use slightly different wording to express the same message.
   2. Example: Trainer says "stay in the correct plane" and 1 minute later while dissecting the same plane says, "stay in the junction"

G. *Trainer repeats Feedback (similar):* trainer gives similar feedback to a previous instance but is now referring to a different stage of the procedure or a different anatomic region
   1. Example: Trainer says "use a sweeping motion to take down those fibers" while the trainee is working on dissecting the left side of the prostate. Thirty minutes later, when the trainee is working on the right side of the prostate the trainer says, "use the sweeping motion here"

H. *Trainer takes over (for safety)*: trainer takes over control of the robot due to concern for patient safety. The trainer is intervening to prevent harm.
   1. Example: Trainee is unable to find the source of bleeding and achieve hemostasis, so the trainer takes over the robot.

I. *Trainer takes over (for non-safety reason)*: trainer takes control of the robot to lend assistance and provide guidance. The trainer is not intervening due to safety concerns.
   1. Examples: the trainer wants to feel the anatomy for themselves and perform a quality control assessment; the trainer takes over to perform a step of the procedure that is beyond the trainee's scope of ability (or that they prefer to personally do); the trainer wants to demonstrate a technique ("show-and-tell")

© 2023 Wong EY et al. *JAMA Network Open.*
