# Supplementary material for: Development of a Classification System for Live Surgical Feedback
Source: JAMA Netw Open. 2023 Jun 28;6(6):e2320702. doi: 10.1001/jamanetworkopen.2023.20702 (PMC10308254; doi:10.1001/jamanetworkopen.2023.20702)
Supplement: Supplement 2. — Data Sharing Statement [file jamanetwopen-e2320702-s002.pdf]

Data Sharing Statement

Wong. Development of a Classification System for Live Surgical Feedback. *JAMA Netw Open*. Published online June 28, 2023. doi:10.1001/jamanetworkopen.2023.20702

Data
Data available: No

Additional Information
Explanation for why data not available: Data are available upon reasonable request.
